# Innate immune deficiencies are associated with severity and poor prognosis in patients with COVID-19

**DOI:** 10.1038/s41598-021-04705-7

**Published:** 2022-01-12

**Authors:** Marine Peyneau, Vanessa Granger, Paul-Henri Wicky, Dounia Khelifi-Touhami, Jean-François Timsit, François-Xavier Lescure, Yazdan Yazdanpanah, Alexy Tran-Dinh, Philippe Montravers, Renato C. Monteiro, Sylvie Chollet-Martin, Margarita Hurtado-Nedelec, Luc de Chaisemartin

**Affiliations:** 1 Autoimmunity and Hypersensitivity Laboratory, AP-HP, Bichat Hospital, 46 Rue Henri Huchard, 75018 Paris, France; 2 INSERM UMR 996, “Inflammation, Microbiome and Immunosurveillance”, Faculty of Pharmacy, Université Paris-Saclay, 92290 Châtenay-Malabry, France; 3 AP-HP, Bichat Hospital Medical and Infectious Diseases ICU (MI2), Paris, France; 4 IAME Université de Paris, Inserm U 1137, Paris, France; 5 Infectious Diseases Department, AP-HP, Hôpital Bichat, Paris, France; 6 Département d’Anesthésie-Réanimation, DMU PARABOL, Université de Paris, AP-HP, Bichat Hospital, Paris, France; 7 INSERM UMR 1152 – ANR10-LABX-17, Paris, France; 8 Center for Research On Inflammation (CRI), Inflamex Laboratory of Excellence, U1149, CNRS ERL8252, Paris, France; 9 Immunological Dysfunction Laboratory, APHP, Bichat Hospital, Paris, France

**Keywords:** Granulocytes, Monocytes and macrophages, Acute inflammation, Viral infection, SARS-CoV-2, Viral host response, Prognostic markers

## Abstract

COVID-19 can cause acute respiratory distress syndrome, leading to death in many individuals. Evidence of a deleterious role of the innate immune system is accumulating, but the precise mechanisms involved remain unclear. In this study, we investigated the links between circulating innate phagocytes and severity in COVID-19 patients. We performed in-depth phenotyping of neutrophil and monocyte subpopulations and measured soluble activation markers in plasma. Additionally, anti-microbial functions (phagocytosis, oxidative burst, and NETosis) were evaluated on fresh cells from patients. Neutrophils and monocytes had a strikingly disturbed phenotype, and elevated concentrations of activation markers (calprotectin, myeloperoxidase, and neutrophil extracellular traps) were measured in plasma. Critical patients had increased CD13^low^ immature neutrophils, LOX-1 + and CCR5 + immunosuppressive neutrophils, and HLA-DR^low^ downregulated monocytes. Markers of immature and immunosuppressive neutrophils were strongly associated with severity. Moreover, neutrophils and monocytes of critical patients had impaired antimicrobial functions, which correlated with organ dysfunction, severe infections, and mortality. Together, our results strongly argue in favor of a pivotal role of innate immunity in COVID-19 severe infections and pleads for targeted therapeutic options.

## Introduction

COVID-19 can cause acute respiratory distress syndrome (ARDS), leading to death in a significant number of individuals^1^. Evidence of a strong involvement of the innate immune system is accumulating^2,3^. High concentrations of proinflammatory cytokines in patients have led to the hypothesis that a “cytokine storm” was one of the main driver of severe COVID-19^4^. While the precise role of cytokines in pathophysiology is debated^5^, their circulating concentrations have been associated with severity^6,7^, and off-label therapeutic blockade of IL-6 and IL-1 have been attempted with some success^8,9^. Furthermore, elevated neutrophil-to-lymphocyte ratio has been linked to severity^10^, and neutrophil infiltration has been described in the lungs and broncho-alveolar lavage fluid (BALF) of infected individuals^11,12^. Complexes of DNA and cytoplasmic proteins secreted by activated neutrophils called Neutrophils Extracellular Traps (NETs) have been detected in patients sera^13^, and are suspected of participating in lung injury and thrombosis associated with severe COVID-19^14,15^. Taking into account these findings and previous knowledge on the role of neutrophils in ARDS^16^, a major involvement of innate immunity in COVID-19 pathophysiology seems likely^17,18^.

Innate immunity is involved in anti-viral responses. Therefore, in addition to their contribution to hyperinflammation, a dysfunction of innate immunity could also contribute to loss of control of viral replication and thus to COVID-19 severity. Recent data from two French consortia show an impaired type I interferon response in COVID-19 patients associated with persistent viral load^19,20^. Moreover, our recent clinical experience and literature report a high incidence (50%) of severe bacterial and fungal infections in non-survivor COVID-19 patients, potentially suggesting an immunosuppression. These infections, in particular invasive aspergillosis, are reminiscent of primary innate immune deficiencies like chronic granulomatous disease^21^. Altogether, these findings strongly suggest a double role for innate cells in hyperinflammatory responses in COVID-19 pathogenesis, but also in the late immunodeficiency observed in some patients. As highlighted by several authors, there is an urgent need to better document the immune response to be able to both enhance its efficacy and prevent its deleterious effects^17^.

In this paper, we investigated the phenotype and functions of circulating phagocytes in severe and critical COVID-19 patients. We found cellular and circulating markers of neutrophil and monocyte activation that were linked to severity. Immature and immunomodulatory subpopulations of innate cells were present in critical patients. At the functional level, phagocytosis, oxidative burst and NETosis were impaired in patients. This functional impairment as well as immature neutrophil markers were associated to organ dysfunction, severe secondary infections, and mortality.

## Methods

All methods were carried out in accordance with relevant guidelines and regulations.

See Supplementary methods for more details.

### Study design

A total of 84 consecutive Covid-19 patients from Bichat Hospital, Paris, France, were included during the first epidemic wave (March-June 2020). Patients were hospitalized in a standard hospital ward in the Infectious Disease Department (non-ICU, n = 40) or in intensive care unit for critically ill patients (ICU patients n = 44). Demographic, clinical, and biological data of patients are summarized in Table 1. Twenty-two healthy blood donors were added to establish the normal range of studied parameters. The study was approved by National Ethics committee “Comité d’Evaluation Ethique de l’INSERM” (CEEI; IRB00003888) under the number 20-715. Informed consent was obtained from all participants.

**Table 1:** Description of the cohort.

	Controls (n = 22)	Mild (n = 40)	Severe (n = 44)	*p*
**Sex**				
Female	14 (64%)	20 (50%)	5 (11%)	** < 0.0001**^**+**^
Male	8 (36%)	20 (50%)	39 (89%)	
**Age (years)**				
Median	49	68	60	** < 0.0001**^**#**^
Mean (SD)	44.5 ± 12.9	69.0 ± 14.9	56.9 ± 10.6	
Range	22–65	44–99	29–79	
**BMI (kg/m**^**2**^**)**				
Median	22.0	25.6	29.9	**0.0088**^**#**^
Mean (SD)	23.6 ± 5.4	28.4 ± 8.0	29.4 ± 5.5	
Range	18.1–38.7	13.3–57.7	16.8–39.1	
Missing data	5 (23%)	13 (33%)	4 (9.1%)	
**Length of hospital stay (days)**				< 0.0001*
Median	–	12.0	34.5	
Mean (SD)	–	14.8 ± 11.7	36.6 ± 18.6	
Range	–	3–64	4–80	
**Deaths**	–	0 (0%)	25 (57%)	** < 0.0001**^**°**^
**Treatments**				
Hydroxychloroquine	–	5 (13%)	12 (27%)	0.1096°
Dexamethasone	–	11 (28%)	34 (77%)	** < 0.0001°**
Lopinavir/Ritonavir	–	7 (18%)	27 (61%)	** < 0.0001°**
Tocilizumab	–	0 (0%)	7 (16%)	**0.0126°**
Anakinra	–	5 (13%)	13 (30%)	0.0674°
Remdesivir	–	0 (0%)	4 (9.1%)	0.1177°
**Pulmonary lesions**				
Mild (< 10%)	–	13 (33%)	2 (4.5%)	**0.0001**^**+**^
Moderate (10–25%)	–	17 (42%)	9 (19%)	
Extensive (25–50%)	–	8 (20%)	12 (27.5%)	
Severe (50–75%)	–	2 (5.0%)	17 (39%)	
Critical (> 75%)	–	0 (0%)	4 (9%)	
**PaO**_**2**_**/FiO**_**2**_				
Median	–	–	166.0	–
Mean (SD)	–	–	210 ± 109	–
Range	–	–	57–466	–
**SAPS II score**				
Median	–	–	60.0	–
Mean (SD)	–	–	38.82 ± 15.1	
Range	–	–	16–81	
**Hospital acquired pneumonia**	–	3 (7.5%)	37 (84%)	** < 0.0001°**
**Time elapsed between FS1 and blood sampling (days)**				
Median	–	16.5	19	0.1357*
Mean (SD)	–	18.2 ± 11.5	22.6 ± 14.1	
Range	–	3–57	4–64	
**Blood count (mean ± SD)**				
Leucocytes (G/L)	6.8 ± 2.2	7.4 ± 4.2	13.2 ± 5.8	** < 0.0001**^**#**^
Neutrophils (G/L)	4.4 ± 1.6	5.1 ± 3.9	11.0 ± 5.7	** < 0.0001**^**#**^
Monocytes (G/L)	0.45 ± 0.15	0.65 ± 0.31	0.74 ± 0.54	0.0861^#^
Lymphocytes (G/L)	1.8 ± 0.5	1.4 ± 0.6	1.2 ± 0.8	**0.0477**^**#**^
Eosinophils (G/L)	0.13 ± 0.14	0.099 ± 0.086	0.17 ± 0.26	0.2190^#^
Basophils (G/L)	0.04 ± 0.02	0.023 ± 0.017	0.030 ± 0.043	0.2151^#^

*Student t-test ; ^+^Chi-square test; °Fisher’s exact test; ^#^ANOVA. FS1 : first day of functional signs.

### Whole blood myeloid cell phenotyping

EDTA-treated blood samples were submitted to multicolor staining for markers of neutrophil and monocyte subpopulations and activation (see Supplementary Table 1 for antibody list). All samples were acquired on a FACS Lyrics cytometer (BD Biosciences), and analysis of data was done on FlowJo 10.0 (this applies to all flow cytometry experiments of this study).

### Phagocytosis assay

Heparinized whole blood was incubated with pH-sensitive pHRodo-conjugated Zymosan bioparticles (ThermoFischer) for 2 h at 37 °C or 4 °C (negative controls). All samples were then subjected to RBC lysis and acquired on a flow cytometer. A phagocytic index was defined as the ratio between pHRodo mean fluorescent intensity between cells at 37 °C and cells at 4 °C.

### Oxidative burst assay

Oxidative burst was measured using redox probe dihydroethidium (600 ng/mL) in heparinized whole blood. Cells were primed with TNFα (5 ng/ml, Bio-techne), LPS (10 ng/ml, Sigma-Aldrich), or TLR7/8 agonist CL097 (2.5 µg/ml, Invivogen) for 45 min at 37° and stimulated with N-formyl-methionyl-leucyl-phenylalanine (fMLF) for 5 min and subjected to RBC lysis (BD BioSciences) and washing before acquisition on a cytometer.

### NETosis assay

Neutrophils were purified from whole blood by negative magnetic selection (Miltenyi Biotech). Purified neutrophils were suspended at 1 × 10^6^ cells/mL in Sytox Green solution (Thermofischer, 2.5 μM) and preincubated for 30 min with either 5 ng/ml TNF-α, 10 ng/ml LPS, 2.5 µg/ml CL097 (Invivogen), or medium. These concentrations are those classically used to prime various neutrophil functions, and they are not sufficient to trigger NET release alone. Then, cells stimulated with either 25 nM phorbol myristate acetate (PMA, Sigma), 1 µM fMLF, or 5 μg/ml *S.aureus* peptidoglycan (PGN, Sigma-Aldrich) for 3 h at 37 °C. DNA release was quantified over time by fluorimetry.

### Quantification of circulating activation markers

Plasma and serum were obtained by centrifugation of fresh blood and immediately frozen at − 80 °C before use. Soluble inflammation parameters CD14 (sCD14), lactoferrin, L-selectin, matrix metallopeptidase 9 (MMP-9), lipocalin (NGAL), IL-8 (CXCL8), myeloperoxidase (MPO) and S100A8/A9 (calprotectin) were quantified in the plasma by multiplex bead assay (Thermofisher Scientific); except for neutrophil elastase that was quantified by ELISA (ThermoFisher Scientific) and Neutrophil Extracellular Traps (NETs) that were quantified by measuring myeloperoxidase (MPO)-DNA complexes using an in-house ELISA^22,23^.

### Statistics

Intergroup differences were analyzed with non-parametric unpaired Mann–Whitney U-test for comparison between two groups and Kruskall-Wallis test followed by Dunn post-test for comparison between more than two groups. Correlation was performed using Spearman correlation. Hierarchical clustering was performed using Euclidian distance and Ward linkage method. Statistical tests were bilateral, and a type I error was fixed at 5%. Statistical analyses were performed with GraphPad Prism versions 9.0 (GraphPad Software Inc.), Statview 5.0 (SAS Institute Inc.) and hierarchical clustering with Genesis 1.8.1 (Gratz University of Technology, https://genome.tugraz.at/genesisclient/genesisclient_description.shtml).

## Results

### Hallmarks of neutrophils and monocytes activation in COVID-19 patients

**Figure 1:**
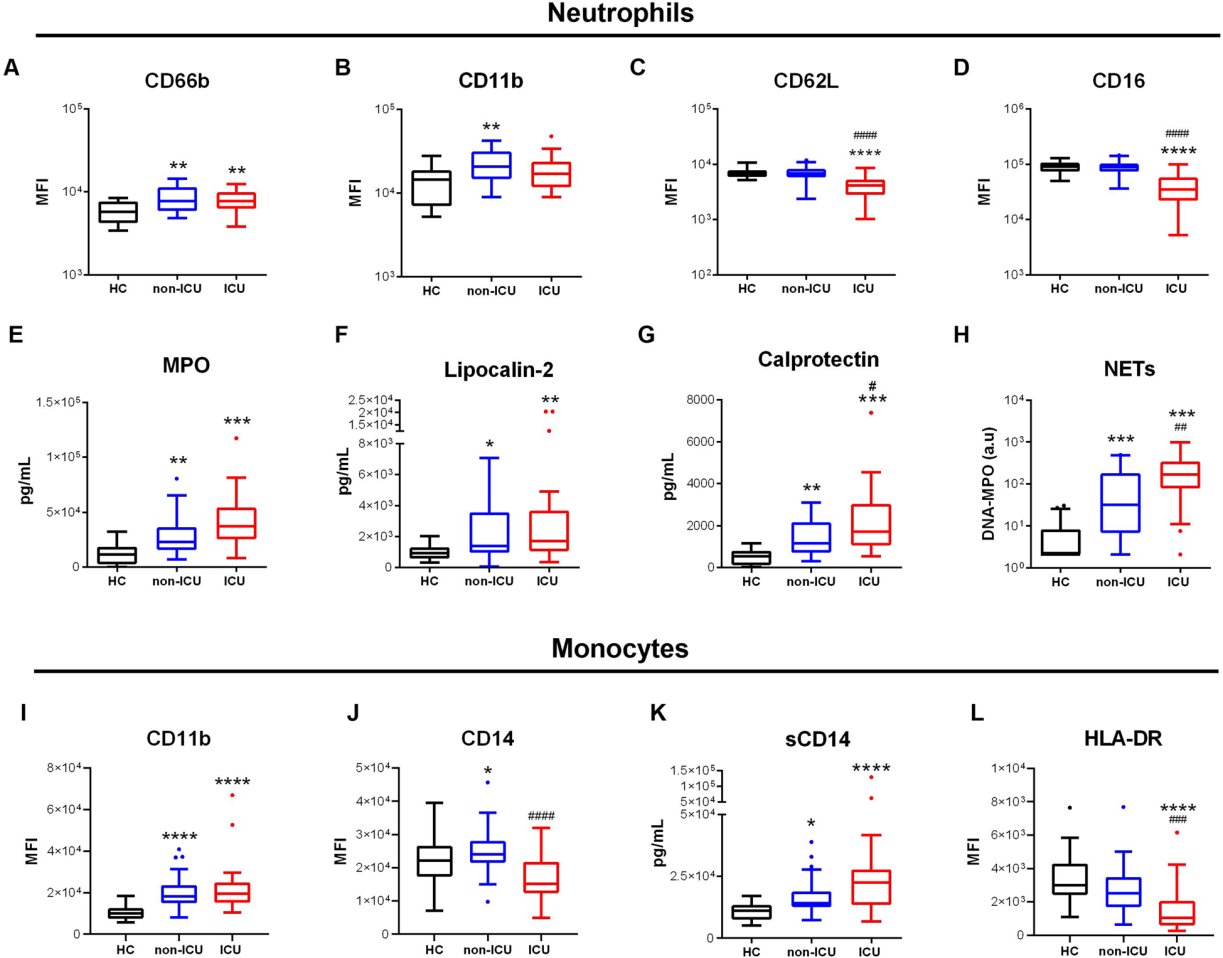
Circulating phagocytes are activated in severe COVID-19. (**A**–**D**) Expression of activation markers CD66b (**A**), CD11b (**B**), CD62L (**C**), CD16 (**D**) on neutrophils by flow cytometry. (**E**–**H**) Concentration in plasma of myeloperoxidase (MPO, **E**), lipocalin-2 (**F**), calprotectin (**G**) and NETs (**H**) in COVID-19 patients and controls. (**I**,**J**) Surface expression of CD11b (**I**) and CD14 (**J**) on monocytes. (**K**) Soluble CD14 concentration in plasma. (**L**) Surface expression of HLA-DR on monocytes. Intergroup comparison by Mann–Whitney U test between healthy controls and ICU or non-ICU patients: ****P < 0.0001, ***P < 0.001, **P < 0.01, *P < 0.05, between non-ICU and ICU patients: ^####^P < 0.0001, ^###^P < 0.001, ^##^P < 0.01, ^#^P < .05. All boxplots whiskers represent 10th and 90th percentiles. *HC* healthy controls.

We first assessed surface and soluble activation markers of blood neutrophils and monocytes in COVID-19 patients (Fig. 1). Neutrophil expression of CD66b and CD11b was significantly elevated in both ICU and non-ICU patients as compared with healthy controls (Fig. 1A,B). Additionally, CD62L and CD16 were strongly downregulated in ICU patients (Fig. 1C,D). Concentrations of MPO, elastase, lipocalin-2, MMP-9, and calprotectin were significantly elevated in both patient groups (Fig. 1E–G, Fig.S1). Moreover, the levels of circulating NETs were strongly increased in both patient groups (Fig. 1H).

Monocytes displayed an activated phenotype with upregulation of CD11b in both patient groups and downregulation of CD14 in ICU patients (Fig. 1I,J). Elevated plasma concentrations of sCD14 were seen in both groups (Fig. 1K). Interestingly, the activation marker HLA-DR was dramatically downregulated in ICU patients, a feature associated with immunoparalysis (Fig. 1L).

Altogether, we show hallmarks of neutrophil and monocyte activation in blood of COVID-19 patients. Moreover, ICU patients exhibit a distinct phenotype with additional neutrophil activation markers and features compatible with downregulated monocytes. This prompted us to explore subpopulations of phagocytes in more details.

### Immunosuppressive and immature neutrophil subsets are increased in COVID-19 patients

**Figure 2:**
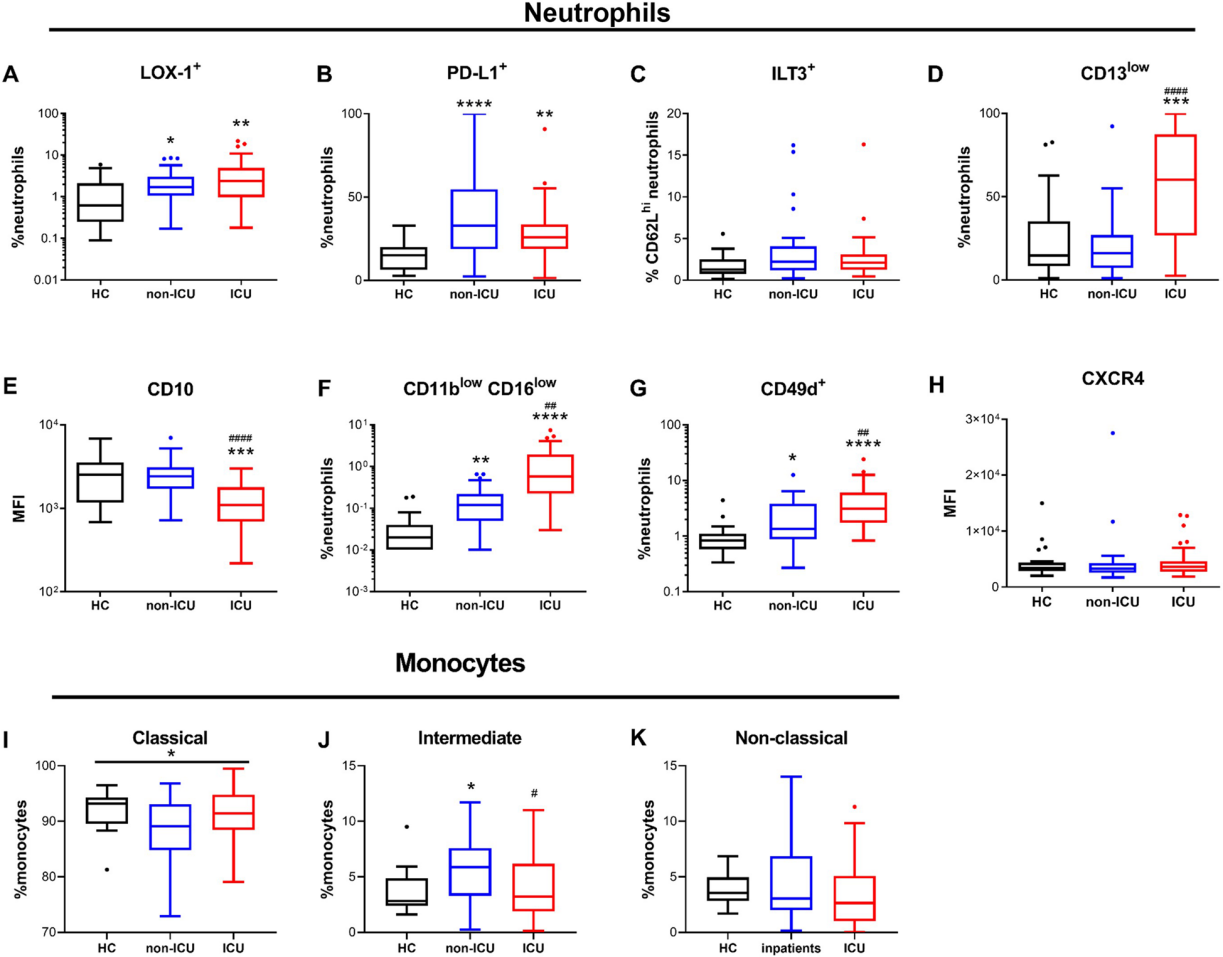
Neutrophil and monocyte subpopulations are disturbed in COVID-19 patients. (**A**–**H**) Percentages of positive cells and surface expression of markers related to subpopulations in neutrophils by flow cytometry. (**A**) LOX-1 + neutrophils, (**B**), PD-L1 + neutrophils, (**C**) ILT3 + CD62L + neutrophils, (**D**) CD13^low^ neutrophils, (**E**) CD10 expression on neutrophils, (**F**) CD11b^low^CD16^low^ neutrophils, (**G**) CD49 + neutrophils, (**H**) HLA-DR expression on neutrophils. (**I**–**K**) Percentages of monocytes subpopulations by flow cytometry. (**I**) CD14 + CD16- classical monocytes, (**J**) CD14 + CD16 + intermediate monocytes, (**K**) CD14^low/-^CD16 + atypical monocytes. Intergroup comparison by Mann–Whitney U test between healthy controls and ICU or non-ICU patients: ****P < 0.0001, ***P < 0.001, **P < 0.01, *P < 0.05, between non-ICU and ICU patients: ^####^P < 0.0001, ^###^P < 0.001, ^##^P < 0.01, ^#^P < .05. All boxplots whiskers represent 10th and 90th percentiles. *HC* healthy controls.

Several kind of neutrophil subpopulations with distinct functions have been described^24^. We found that percentages of LOX-1 + , CCR5^hi^ and PD-L1 + immunosuppressive neutrophils were significantly higher in both patient groups as compared to healthy controls (Fig. 2A,B).

In contrast, no difference was seen in the percentage of ILT3 + cells between the 3 groups (Fig. 2C). CD13^low^ and CD10^low^ immature neutrophil populations were dramatically increased in ICU patients as compared to both non-ICU patients and healthy controls (Fig. 2D,E). Additionally, CD11b^low^CD16^low^ cells, another immature neutrophil population^25^ was also increased in both non-ICU and ICU patients as compared to healthy controls (Fig. 2F), as well as lung inflammation-related CD49 + neutrophils^26^ (Fig. 2G). Finally, CXCR4^hi^ cells described as "senescent" neutrophils^27,28^ did not differ between patients and controls (Fig. 2H).

We next measured the proportion of monocytes subpopulations (classical CD14^+^/CD16^-^, intermediate CD14^+^/CD16^+^, and non-classical CD14^low^/CD16^+^). Compared to healthy controls, only intermediate monocytes were significantly higher in non-ICU patients (Fig. 2I–K).

All in all, we demonstrate disturbed subpopulation distribution in both neutrophils and monocytes in COVID-19 patients, which was more pronounced in ICU patients. The increase in immunosuppressive and immature neutrophils in ICU patients is evocative of an impaired immune response. Therefore, we addressed how these phenotypes were associated with patients’ outcome.

### Markers of immature and immunosuppressive phagocytes are associated with severity and poor outcome

**Figure 3:**
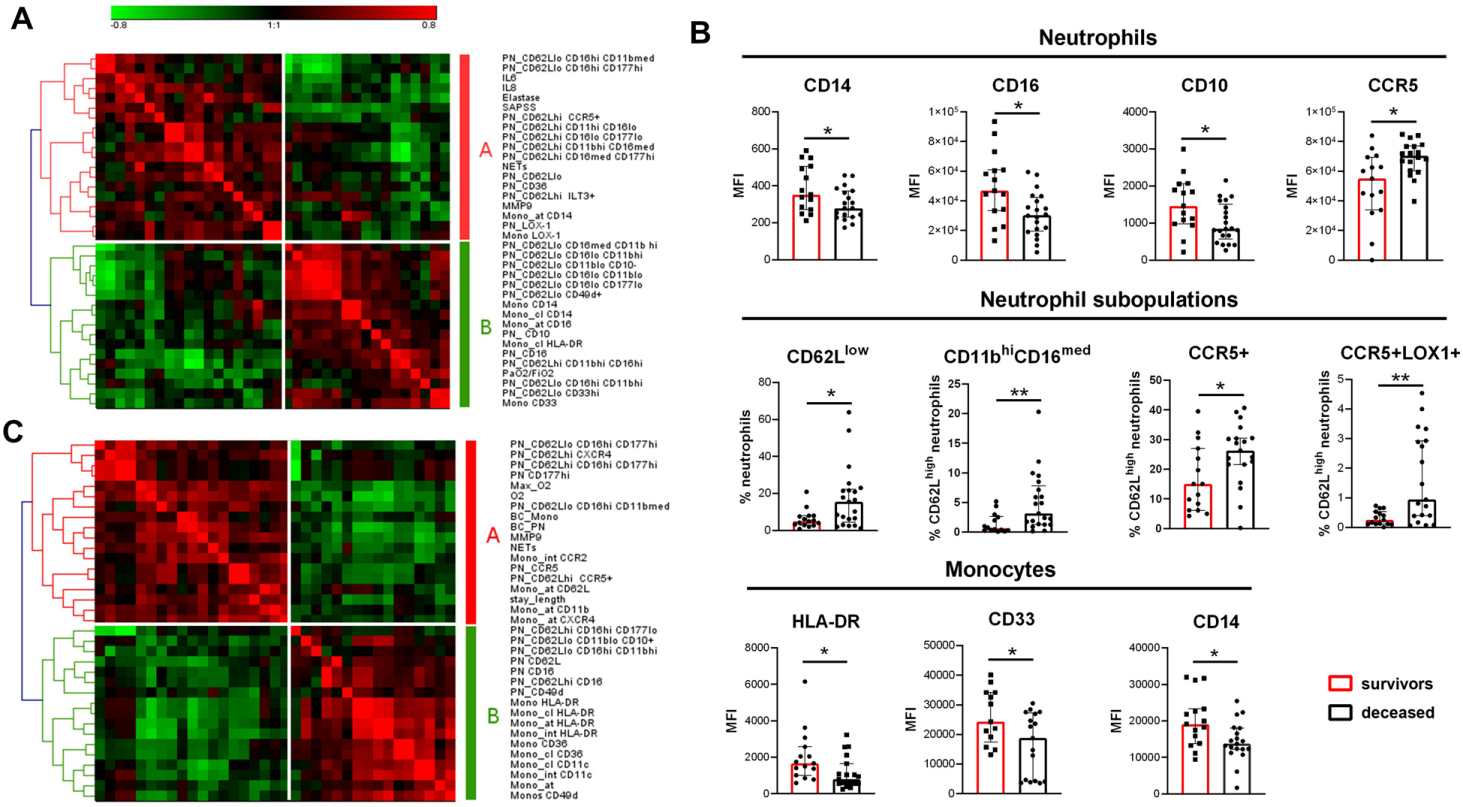
Association of innate immunity-related markers with severity. (**A**) Hierarchical clustering of the correlation matrix from phenotypic and soluble markers significantly correlated with SAPS II score or the PaO_2_/FiO_2_ ratio in ICU patients. Red is for positive correlation and green for negative correlation. Intensity of color is proportional to Spearman correlation coefficient. Cluster A groups markers with a correlation profile similar to SAPS II score. Cluster B groups markers with correlation profiles similar with PaO_2_/FiO_2_ ratio. (**B**) Markers significantly different between patient who survived (red) or died (black) during their ICU stay. The histograms represent the median and the error bars the interquartile range. Intergroup comparison by Mann–Whitney U test between survivors and deceased patients, **P < 0.01, *P < 0.05. (**C**) Hierarchical clustering of the correlation matrix from phenotypic and soluble markers significantly correlated with external oxygen requirement on the day of sampling (O_2_), maximal external oxygen requirement (Max_O2) or length of hospitalization in non-ICU patients (stay_lengh). Red is for positive correlation and green for negative correlation. Intensity of color is proportional to Spearman correlation coefficient. Cluster A groups markers with a correlation profile similar to O2, Max_O2 and stay_length. Cluster B groups markers negatively correlated to cluster A. BC_PN: neutrophil absolute count; BC_Mono: monocyte absolute count. Hierarchical clustering was made with Genesis 1.8.1 (Gratz University of Technology, https://genome.tugraz.at/genesisclient/genesisclient_description.shtml).

To evaluate the links between phenotypic changes with disease severity and prognosis, we computed correlations with relevant clinical data in ICU and non-ICU patients separately to avoid population bias (see Table 1). In ICU patients, severity was assessed by SAPS II, the PaO_2_/FiO_2_ ratio, and mortality (Fig. 3A,B, Fig. S2). A hierarchical clustering of significant correlations highlighted a set of variables correlating with SAPS II and therefore linked to severity (cluster A, Fig. 3A). These variables contained parameters reflecting inflammation (IL-6 and IL-8 concentrations), as well as neutrophil activation, e.g. plasma concentrations of NETs, elastase, MMP9, and increased CD62L^low^ subpopulation. We also found markers associated with immunosuppressive neutrophils like high expression of LOX-1, and CCR5. On the contrary, variables negatively associated with SAPS II (cluster B) contained expression of maturity markers CD10 and CD16, and monocyte expression of HLA-DR, CD14, and CD33.

These findings were confirmed by the analysis of markers associated with mortality in ICU patients (Fig. 3B, Fig. S2). Neutrophils of deceased patients had significantly lower CD16 and CD10 expression, increased CCR5 expression, and higher proportion of CD62L^low^ and CD11b^hi^ CD16^med^ activated and CCR5 + LOX-1 + immunosuppressive neutrophil subpopulation. Monocytes from deceased patients had lower expression of HLA-DR, CD33 and CD14.

Finally, circulating concentrations of IL-6 and neutrophil elastase were significantly higher in deceased patients (Fig. S2).

In the group of non-ICU patients, no unfavorable outcome was recorded during the study time (i.e., no death or ICU admission). We therefore assessed severity by exogenous oxygen (O_2_) requirement and duration of stay in the hospital (Fig. 3C). As for ICU patients, we identified a cluster of variables correlated with severity (higher O_2_ requirement and longer stay; cluster A), which contained neutrophil activation markers like circulating MMP9 and NETs, and low expression of CD62L. Higher expression of CCR5 and lower CD16 on neutrophils as well as lower HLA-DR on monocytes were also linked to severity.

Globally, our analyses show significant associations of severity and poor prognosis with markers of neutrophil activation, higher CCR5 and lower CD16 expression on neutrophils, and HLA downregulation on monocytes in both ICU and non-ICU patients groups.

Since some of these features are associated with activated phagocytes, but other to suppressive or immature and therefore less active cells, we next assessed what was the resulting effect of these phenotypical features on the functions of innate cells isolated from the patients.

### Phagocytes of COVID-19 patients display impaired anti-microbial functions

**Figure 4:**
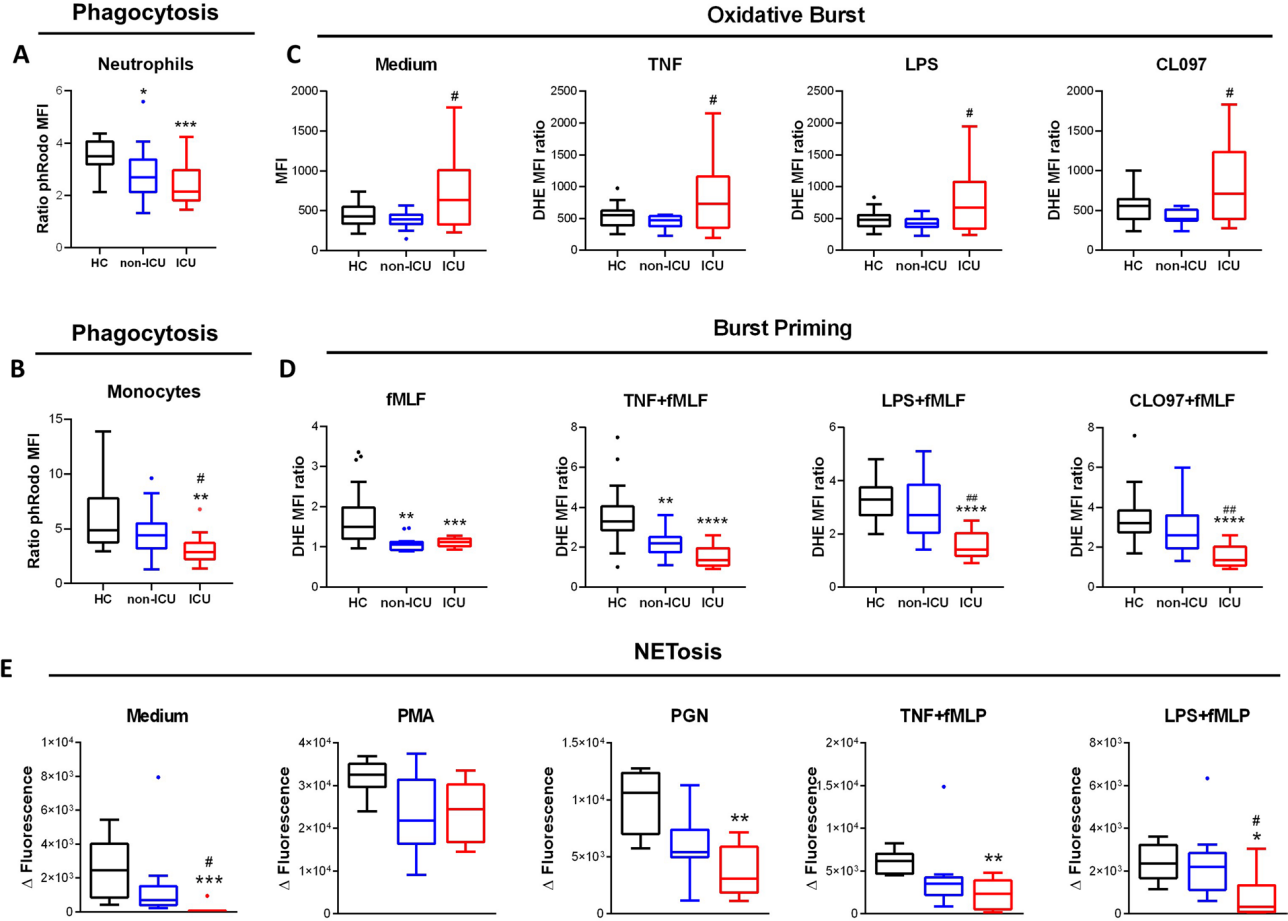
Anti-microbial functions of circulating phagocytes are impaired in severe COVID-19. (**A**,**B**) Measurement of phagocytosis capacity by phRodo-conjugated Zymosan particles uptake in (**A**) neutrophils and (**B**) monocytes. (**C**,**D**) Measurement of (**C**) oxidative burst in response to medium, Tumor Necrosis Factor alpha (TNF), lipopolysaccharide (LPS), TLR8 ligand CLO97, and (**D**) priming of formyl-methionine-leucine-phenylalanine (fMLF)-induced burst by TNF, LPS and CLO-97 in neutrophils. (**E**) Measurement of neutrophils NETosis capacity in response to medium, phorbol myristate acetate (PMA), peptidoglycan (PGN), TNF and fMLF, LPS and fMLF. Intergroup comparison by Mann–Whitney U test between healthy controls and ICU or non-ICU patients, ****P < 0.0001, ***P < 0.001, **P < 0.01, *P < 0.05., between non-ICU and ICU patients, ^##^P < 0.01, ^#^P < .05. All boxplots whiskers represent 10th and 90th percentiles.

We tested canonical myeloid anti-bacterial functions (phagocytosis, oxidative burst and NETosis) on neutrophils and monocytes freshly isolated from patients (Fig. 4). We show that phagocytosis was significantly impaired in both neutrophils and monocytes from ICU and non-ICU patients (Fig. 4A,B), resulting in a lower ability to clear pathogens.

Neutrophils from ICU patients showed higher reactive oxygen species (ROS) production as steady state that non-ICU patients and healthy controls at baseline and in response to priming agents (TNFα, LPS, TLR8 agonist CLO97) and weak burst agonist formyl-methionine-leucin-phenylalanine (fMLF, Fig. 4C). Additionally, burst priming by combining fMLF with priming agents was significantly lower in ICU patients, and even in non-ICU patients for fMLF alone and TNFα priming condition (Fig. 4D). Altogether, these results suggest that neutrophils are hyperactivated at basal state in ICU patients but exhibit a decreased capacity to be reactivated, suggesting higher susceptibility to bacterial or fungal infections. Finally, we measured the NETosis capacity of freshly isolated neutrophils from COVID-19 patients (Fig. 4E). Spontaneous DNA release (in the absence of stimulus) or in response to various known physiological NETosis triggers like TLR2 ligand peptidoglycan (PGN) or fMLF combined with TNFα or LPS was significantly decreased in ICU patients.

Altogether, these functional results strongly suggest that neutrophils and monocytes of severe COVID-19 patients are dysfunctional and exhibit a decreased capacity to fight against pathogens. Such impaired functions are likely to have an impact on host immune response and thus to be detrimental for the patient. Therefore, we measured the association of phagocyte functionality with disease severity and prognosis.

### Neutrophil and monocyte dysfunctions are associated with poor prognosis in ICU patients

**Figure 5:**
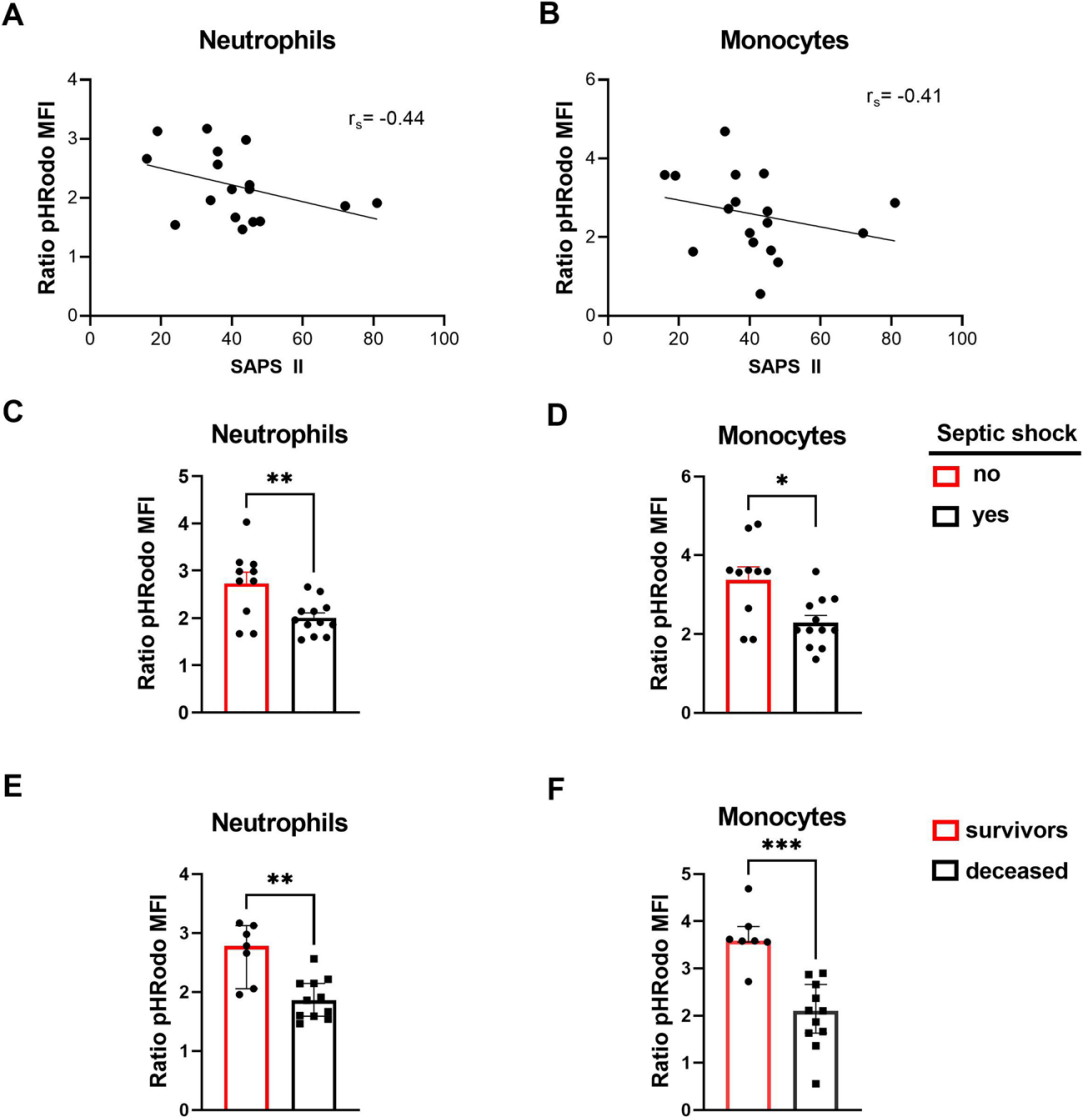
Functional impairment of phagocytes is linked to organ dysfunction, septic shock, and mortality in ICU patients. (**A**,**B**) Correlation between phagocytosis and SAPS II in (**A**) neutrophils and (**B**) monocytes. *Rs* Spearman correlation coefficient Rho. (**C**,**D**) Phagocytosis capacity in patients with (black) or without (red) a septic shock of (**C**) neutrophils and (**D**) monocytes. The histograms represent the median and the error bars the interquartile range**.** Intergroup comparison by Mann–Whitney U test , **P < 0.01, *P < 0.05. **(E–F)** Phagocytosis capacity in survivors (red) and deceased (black) ICU patients of (**E**) neutrophils and (**F**) monocytes. Intergroup comparison by Mann–Whitney U, ***P < 0.001, **P < 0.01.

To quantify neutrophil and monocyte antibacterial functions we chose phagocytosis as an indicator since it involves several cellular mechanisms and was decreased in both patient groups (Fig. 5).

Phagocytosis impairment was linked to organ dysfunction as it correlated negatively with SAPS II score for both cell types (Fig. 5A,B). Additionally, patients who presented with septic shock had significantly less phagocytic activity that patients that did not (Fig. 5C,D). Finally, phagocytosis capacity was much lower in non-survivor patients as compared to survivors. (Fig. 5E,F).

We thus conclude that neutrophil and monocyte functional impairment in COVID-19 patients is linked to severity and poor outcome, at least in part by favoring the occurrence of severe infections.

## Discussion

In this study, we investigated the links between circulating innate phagocyte phenotype and functions and severity in COVID-19 patients. We found cellular and molecular features of myeloid cell activation and disturbed subpopulation distribution in both ICU and non-ICU patients. However, ICU patients presented a distinct set of parameters suggestive of immunosuppressive and immature neutrophils that clearly distinguished them from non-ICU patients. NETs and MMP-9 plasma concentrations, overexpression of CCR5 and downregulation of CD16 and CD62L on neutrophils, as well as downregulation of HLA-DR on monocytes were associated with severity and prognosis in both non-ICU and ICU patients. Overexpression of LOX-1 and downregulation of CD10 on neutrophils, were linked to severity in ICU patients only, while overexpression of CXCR4 and downregulation of CD49d on both cell types was detrimental in non-ICU patients. Most importantly, we show by exploring several cellular functions that neutrophils and monocytes from both patient groups had impaired antimicrobial mechanisms and that this loss of function was linked to organ dysfunction, severe infections, and mortality.

Activated myeloid cells are detrimental in various adult lung diseases, in particular in ARDS^29,30^. We found several circulating and membrane activation markers of neutrophils linked to severity and prognosis in COVID-19 patients. This is in line with large-scale studies that have correlated basic immune parameters and outcome in COVID-19 patients^10,31,32^. However, none of these studies separated ICU from non-ICU patients, which is ground for numerous biases since both patient populations significantly differ for sex, age, BMI, and treatment received. Here we calculated correlations between immune and clinical parameters in each patient group separately. Interestingly, some parameters were associated with severity in both patient groups, most of them being neutrophil activation markers (e.g. NET or MMP9 concentrations, or downregulation of surface CD62L and CD16), which advocates for a globally deleterious role of neutrophils in both ICU and non-ICU patients. Other markers, like CCR5 overexpression, have been shown to be associated with immunosuppressive neutrophils and lower survival in several cancer reports^33^, which could imply that these cell play a role in suppression of adaptive immune response in COVID an explain their link with poorer prognosis. In the same line of thought, HLA-DR downregulation on monocytes is a marker known to be linked to immunoparalysis and severity in sepsis^34,35^. Variables linked to severity in ICU patients included increased IL-6 and IL-8 concentrations, expression of LOX-1, downregulation of CD10 and elevated neutrophil elastase concentrations. LOX-1 is a receptor to oxidized lipids and has been shown to play a role in neutrophil recruitment to the lungs^36^, but also identifies suppressor neutrophils in cancer^37^, two features susceptible to play a major role in COVID pathophysiology. CD10 is a maturity marker, and low CD10 expression has been associated with immature and less functional neutrophils^38,39^. Finally, neutrophil elastase enzymatic activity is strongly suspected to participate in lung injury during ARDS^40^, and is also able to impair adaptive immune responses and facilitate viral entry^41,42^. Thus, compared with non-ICU patients, ICU patients present additional features of proinflammatory immune components but also immature and immunosuppressive cells that contribute to global immune dysfunction and may explain their more severe condition.

To understand the role of blood myeloid cells in COVID-19 pathogenesis, it is important to evaluate not only the populations involved but also their functionality. We tested typical myeloid cell functions (phagocytosis, oxidative burst, NETosis), and found that cells from COVID patients were less functional than cells from healthy controls. While we used microbial components and not live microorganisms, we chose a range of stimuli representing major neutrophil microbial activating receptors (TLR2, TLR4, and Dectin-1), as well as cytokines. Neutrophil functions were decreased in all cases, making a dysfunction in response to live micro-organism likely. Neutrophil impairment was much more pronounced in ICU patients, even if some level of dysfunction could be seen in non-ICU patients. While this may seem paradoxical in regard to the elevated expression of activation markers that we and others have described, this is similar to the immunoparalysis setting described in severe sepsis^43^. In this setting, neutrophils have defective oxidative burst and phagocytosis despite extremely high expression of cellular and soluble activation markers^43,44^. This global neutrophil dysfunction could be due to a massive arrival of immature neutrophils (emergency granulopoiesis), or to an inhibitory effect of dysregulated inflammation. However, in both cases, our findings suggest that in severe COVID-19, not only neutrophil and monocyte activation by-products are deleterious, but also that innate immune response is impaired. The links we found between innate immune dysfunction, disease severity and mortality strongly suggest a pivotal role for innate phagocytes in loss of control of the infection and ultimately, in disease outcome.

There are some limitations to this study. First, this study was performed in the middle of the first wave of the COVID-19 pandemic in France, when many treatments were tested in parallel on critical patients, increasing variability between patients and reducing our ability to detect small effects. In the same line of thought, immunomodulatory treatments were used either on almost all patients (e.g. corticosteroids) or in a small number of patients for each molecules (e.g. tocilizumab), which precludes from in-depth multivariate statistical analysis that could control for their use. Finally, it was not possible for logistic reasons to realize a longitudinal sampling of the patients, which prevent us to reconstitute the precise trajectory of immune responses.

Despite these limitations, we were able to see some strongly significant associations that we are in the process of confirming on a larger cohort from the second and third wave of infections.

In conclusion, we found that innate immune deficiency is present in COVID-19 patients and associated with disease severity and prognosis. These findings indicate that SARS-CoV2 infection may induce an overactivation of the innate system resulting in apparition of abnormal subpopulations and exhaustion of cellular effectors. Such dysregulation of innate responses strongly advocates for the use of systematic antibiotic and antifungal prophylaxis in severe patients, and warrants further studies to assess efficacy of neutrophil-related therapeutics like elastase inhibitors, and the usefulness of severity-associated markers as prognosis tools in COVID-19.

## Supplementary Information

Supplementary Information 1.

Supplementary Figures.

Supplementary Table 1.
