# Supplementary material for: Innate immune deficiencies are associated with severity and poor prognosis in patients with COVID-19
Source: Sci Rep. 2022 Jan 12;12:638. doi: 10.1038/s41598-021-04705-7 (PMC8755788; doi:10.1038/s41598-021-04705-7)
Supplement: Supplementary file 1 — Supplementary Information 1. [file 41598_2021_4705_MOESM1_ESM.docx]

Innate immune deficiencies are associated with severity and poor prognosis in patients with COVID-19

**Authors:** Marine Peyneau^1,2^, Vanessa Granger^1,2^, Paul-Henri Wicky^3,4^, Dounia Khelifi-Touhami^1^, Jean-François Timsit^3,4^, François-Xavier Lescure^4,5^, Yazdan Yazdanpanah^4,5^, Alexy Tran-Dihn^6,7^, Philippe Montravers^6,7^, Renato C. Monteiro^8,9^, Sylvie Chollet-Martin^1,2^, Margarita Hurtado-Nedelec^8,9^, Luc de Chaisemartin^1,2^*

**Supplementary Material**

**Supplementary Methods**

**Study design**

A total of 84 consecutive Covid-19 patients from Bichat Hospital, Paris, France, were included during the first epidemic wave (March-June 2020). Patients were hospitalized in a standard hospital ward in the Infectious Disease Department (non-ICU, n=40) or in intensive care unit for critically ill patients (ICU patients n=44). Demographic, clinical, and biological data of patients are summarized in Table 1. Twenty-two healthy blood donors were added to establish the normal range of studied parameters. The study was approved by National Ethics committee CEEI/IRB under the number 20-715.

**Whole blood myeloid cell phenotyping**

EDTA-treated blood samples were incubated with several antibody combinations containing a wide selection of markers for neutrophil and monocyte subpopulations and activation (see Supplemental Table 1 for antibody list). After 30 min at 4°C, samples were subjected to red blood cell (RBC) lysis (FACS Lysing solution, BD BioSciences) for 10 min and washed before acquisition. All samples were acquired on a FACS Lyrics cytometer (BD Biosciences), and analysis of data was done on FlowJo 10.0 (this applies to all flow cytometry experiments of this study).

**Phagocytosis assay**

Heparinized whole blood was incubated with pH-sensitive pHRodo-conjugated Zymosan bioparticles (ThermoFischer) for 2 hours at 37°C in a water bath under mild shaking. Control samples were incubated with the same amount of bioparticles and for the same time at 4°C to inhibit phagocytosis. All samples were then subjected to RBC lysis. After washing, cells were acquired on a FACS Lyrics cytometer. Results are expressed as the ratio of pHRodo mean fluorescent intensity (MFI) from 37°C-incubated samples to 4°C-incubated control samples.

**Oxidative burst assay**

Diluted heparinized whole blood (1/10) was pre-incubated with 600ng/ml dihydroethidium (DHE, Sigma-Aldrich) probe for 15 min at 37°C under agitation in a water bath before being primed with TNFα (5ng/ml, Bio-techne), LPS (10ng/ml, Sigma-Aldrich), or TLR7/8 agonist CL097 (2.5µg/ml, Invivogen) for 45 min at 37°c. Samples were then stimulated with N-formyl-methionyl-leucyl-phenylalanine (fMLF 1µM, Sigma-Aldrich) for 5 min and subjected to RBC lysis (BD BioSciences) and washing before acquisition on a FACS Lyrics cytometer. Results were expressed in MFI and a stimulation index (SI) was calculated (MFI ratio between stimulated and unstimulated cells).

**NETosis assay**

Neutrophils were purified from EDTA-treated whole blood using MACSXpress Neutrophil isolation kit (Miltenyi Biotech) which allowed a purity routinely over 98%. Purified neutrophils were suspended at 1x10^6^ cells/mL in Sytox Green solution (Thermofischer, final concentration 2.5μM) diluted in Hank’s Balanced Salt (HBSS) and preincubated for 30 min with either 5 ng/ml TNF-α, 10 ng/ml lipopolysaccharide (LPS, Sigma) , 2.5µg/ml CL097 (Invivogen), or medium. Then, 150μL of cells were seeded in black 96-well plates and stimulated with either 25nM phorbol myristate acetate (PMA, Sigma), 1µM fMLF, or 5μg/ml *S.aureus* peptidoglycan (PGN, Sigma-Aldrich) for 3h at 37°C. Cell-free DNA release was quantified over time on an Infinite 200 PRO microplate reader (TECAN). Results were expressed as the increase in fluorescence over 3h normalized for baseline.

**Quantification of circulating activation markers**

Plasma and serum were obtained by centrifugation of fresh blood and immediately frozen at -80°C after aliquoting.

Soluble CD14 (sCD14), lactoferrin, L-selectin, matrix metallopeptidase 9 (MMP-9), neutrophil gelatinase-associated lipocalin (NGAL), IL-8 (CXCL8), myeloperoxidase (MPO) and S100A8/A9 (calprotectin) were quantified in the plasma using Procartaplex Multiplex Immunoassay (Thermofisher Scientific) according to the manufacturer’s instructions. Plates were read on a MAGPIX System (Merck Millipore) analyzer and data analyzed with Milliplex software. Human neutrophil elastase plasma levels were quantified by ELISA using Human PMN-Elastase ELISA kit (ThermoFisher Scientific) according to manufacturer’s instructions. Absorbance was read at 450nm using a Multiskan FC spectrophotometer (ThermoFischer).

Neutrophil Extracellular Traps (NETs) were quantified by measuring myeloperoxidase (MPO)-DNA complexes in serum samples using an in-house capture ELISA already described ^61,62^. Samples were interpolated from a standard curve and results were expressed in arbitrary units (a.u.). The detection range was 4.2-724 a.u. Values below the lower limit of detection (LDD) were assigned the LDD/2 (i.e. 2.1 a.u.) and values above the maximal of standard curve (MSC) were assigned the 1.5 X MSC (i.e. 1086 a.u).

**Statistics**

Intergroup differences were analyzed with non-parametric unpaired Mann-Whitney U-test for comparison between two groups and Kruskall-Wallis test followed by Dunn post-test for comparison between more than two groups. Correlation between continuous data was performed using Spearman correlation. Hierarchical clustering was performed using Euclidian distance and Ward linkage method. Statistical tests were bilateral, and a type I error was fixed at 5%. Statistical analyses were performed with GraphPad Prism versions 8.0 (GraphPad Software Inc.), Statview 5.0 (SAS Institute Inc.) and hierarchical clustering with Genesis 1.8.1 (Gratz University of Technology).
