# Supplementary material for: Innate immune deficiencies are associated with severity and poor prognosis in patients with COVID-19
Source: Sci Rep. 2022 Jan 12;12:638. doi: 10.1038/s41598-021-04705-7 (PMC8755788; doi:10.1038/s41598-021-04705-7)
Supplement: Supplementary file 2 — Supplementary Figures. [file 41598_2021_4705_MOESM2_ESM.pptx]

## Slide 1
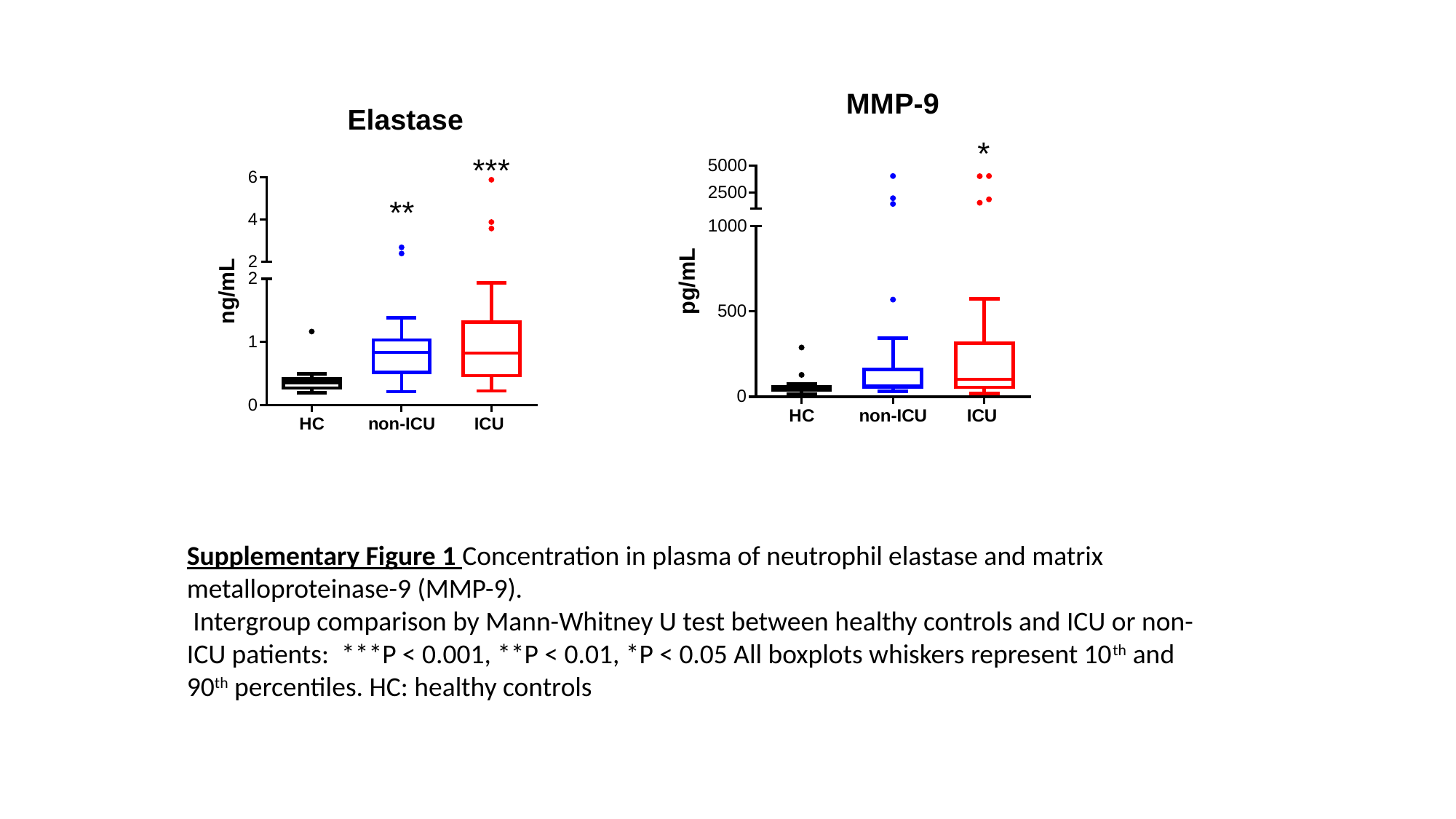

Supplementary Figure 1 Concentration in plasma of neutrophil elastase and matrix metalloproteinase-9 (MMP-9).
 Intergroup comparison by Mann-Whitney U test between healthy controls and ICU or non-ICU patients: ***P < 0.001, **P < 0.01, *P < 0.05 All boxplots whiskers represent 10th and 90th percentiles. HC: healthy controls

## Slide 2
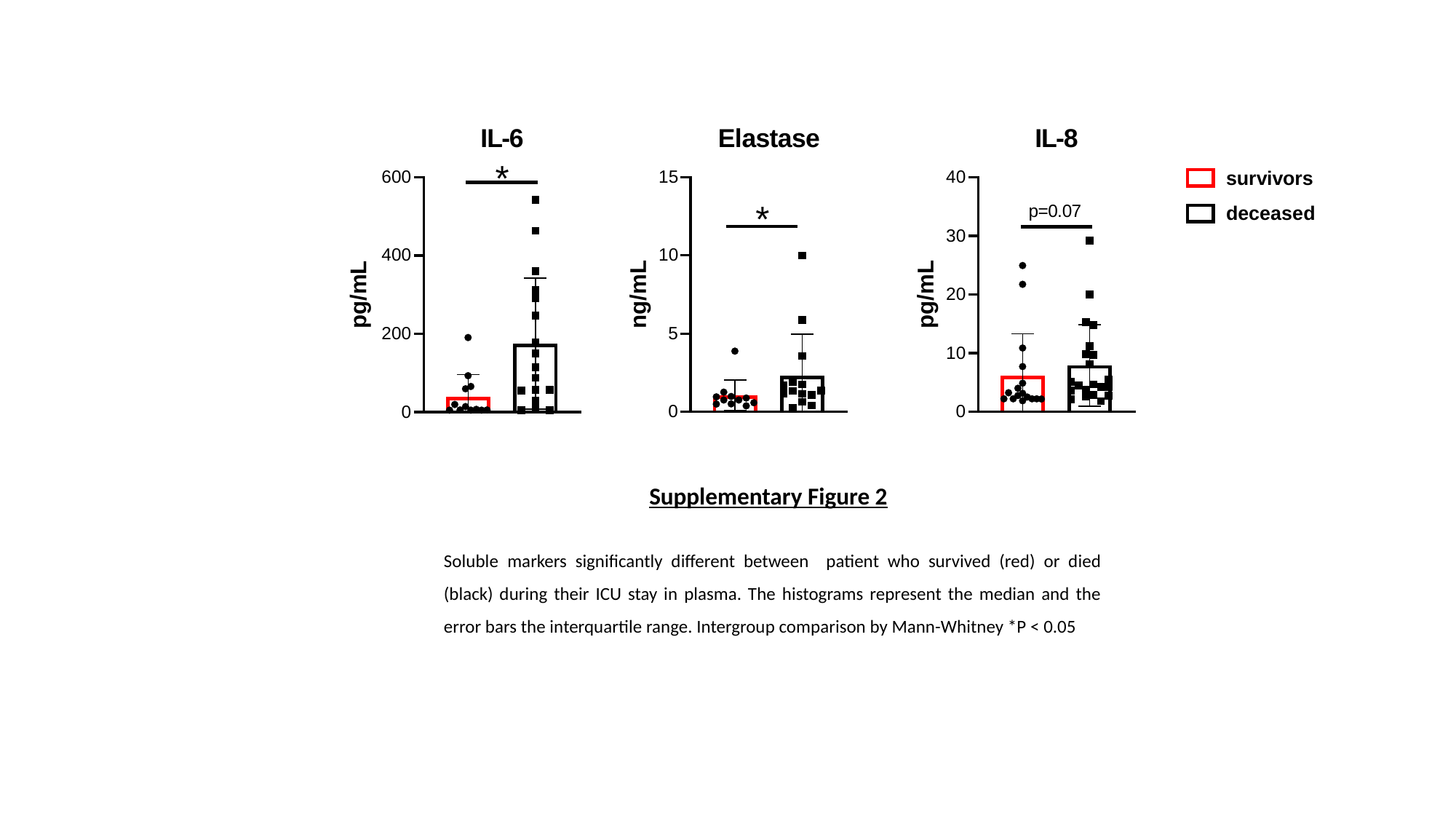

# Supplementary Figure 2
Soluble markers significantly different between patient who survived (red) or died (black) during their ICU stay in plasma. The histograms represent the median and the error bars the interquartile range. Intergroup comparison by Mann-Whitney *P < 0.05
