# Supplementary material for: Innate immune deficiencies are associated with severity and poor prognosis in patients with COVID-19
Source: Sci Rep. 2022 Jan 12;12:638. doi: 10.1038/s41598-021-04705-7 (PMC8755788; doi:10.1038/s41598-021-04705-7)
Supplement: Supplementary file 3 — Supplementary Table 1. [file 41598_2021_4705_MOESM3_ESM.docx]

| **Target** | **Clone** | **Provider** |
| --- | --- | --- |
| **CD66b** | G10F5 | Biolegend |
| **CD11b** | ICRF-44 | BD Biosciences |
| **CD62L** | DREG-56 | BD Biosciences |
| **CD16** | 3G8 | Biolegend |
| **CD14** | MφP9 | BD Biosciences |
| **HLA-DR** | G46-6 | BD Biosciences |
| **LOX-1** | 15C4 | Biolegend |
| **PD-L1** | MIH3 | Biolegend |
| **CD10** | HI10a | Biolegend |
| **CD13** | WM15 | Biolegend |
| **ILT3** | ZM4.1 | Biolegend |
| **CD49d** | 9F10 | Biolegend |
| **CD195** | J418F1 | Biolegend |
| **CD184** | 12G5 | Biolegend |
| **CD33** | WM53 | Biolegend |
| **CD177** | MEM-166 | Biolegend |
| **CD11c** | S-HCL-3 | Biolegend |
| **CCR2** | K036C2 | Biolegend |
| **CD36** | 5-271 | Biolegend |

**Supplementary Table 1**

Antibody list
